# Transoral Tongue Suspension for Obstructive Sleep Apnea—A Preliminary Study

**DOI:** 10.3390/jcm11174960

**Published:** 2022-08-24

**Authors:** Li-Jen Hsin, Yi-Chan Lee, Wan-Ni Lin, Yi-An Lu, Li-Ang Lee, Ming-Shao Tsai, Wen-Nuan Cheng, Yen-Ting Chiang, Hsueh-Yu Li

**Affiliations:** 1 Departments of Otolaryngology-Head & Neck Surgery, Chang Gung Memorial Hospital, Linkou Main Branch, Taoyuan 33305, Taiwan; 2 College of Medicine, Chang Gung University, Taoyuan 33302, Taiwan; 3 Departments of Otolaryngology-Head & Neck Surgery, Chang Gung Memorial Hospital, Keelung Branch, Keelung 20401, Taiwan; 4 Departments of Otolaryngology-Head & Neck Surgery, Chang Gung Memorial Hospital, Chayi Branch, Chayi 61363, Taiwan; 5 Graduate Institute of Clinical Medical Sciences, College of Medicine, Chang Gung University, Taoyuan 33302, Taiwan; 6 Health Information and Epidemiology Laboratory, Chang Gung Memorial Hospital, Chiayi 61363, Taiwan; 7 Department of Sports Sciences, University of Taipei, Taipei 11153, Taiwan

**Keywords:** transoral tongue suspension, obstructive sleep apnea, tongue base suspension, uvulopalatopharyngoplasty, snoring, daytime sleepiness, sleep surgery

## Abstract

Objectives: To evaluate the safety and efficacy of a novel technique for transoral tongue suspension (TOTS) in obstructive sleep apnea (OSA) patients. Material and Methods: The retrospective study enrolled 24 consecutive OSA patients (21 males; average age, 43 years; average apnea–hypopnea index (AHI), 42.2 event/h; average body mass index (BMI), 25.7 kg/m^2^) with tongue obstruction confirmed by drug-induced sleep endoscopy. All patients received TOTS as the main procedure in conjunction with uvulopalatopharyngoplasty (UPPP). Key procedures of TOTS included a transoral sublabial approach, drilling two holes on the mandible, passing the polypropylene through the hole to the tongue base using a suture passer and returning the polypropylene through loop traction, and tying the polypropylene to the mandible. Lingual tonsil ablation (*n* = 8) was also implemented in hypertrophic lingual tonsils (grades III and IV). Results: The operation time for TOTS was around 30 min. No wound bleeding or airway compromise occurred throughout the postoperative period. Minor complications were temporary and included swelling of the tongue, numbness of the lower incisor, and sublabial wound dehiscence (*n* = 2). The quality of life improved significantly in the patients’ subjective daytime sleepiness according to the Epworth Sleepiness Scale (11.4 ± 3.2 vs. 5.7 ± 1.6, *p* < 0.001). The objective parameters of OSA also improved significantly in the apnea/hypopnea index (42.2 ± 21.8 vs. 19.5 ± 16.2, *p* < 0.001), minimal oxygen saturation (77.1 ± 12.2 vs. 81.7 ± 8.1, *p* = 0.026), and snoring index (207 ± 141 vs. 101 ± 91, *p* = 0.03). Conclusions: The demonstrated TOTS showed its advantage in low morbidity with a scarless exterior and easy performance with free availability in treating adult OSA patients with tongue obstruction. TOTS combined with UPPP significantly improved AHI and daytime sleepiness. TOTS can be implemented with lingual tonsillectomy to achieve both stabilization of the tongue and widening of hypopharyngeal airway.

## 1. Introduction

Obstructive sleep apnea (OSA) is defined as repeated episodes of obstructive apnea and hypopnea during sleep [1]. The epidemiology of clinically significant OSA in the United States and Europe has been reported to affect 14% and up to 49% of middle-aged men, causing a higher cardiovascular risk and death [2]. OSA is a multifactorial disease involving anatomy, muscle tone, arousal, and loop [3,4]. Airway obstruction in OSA patients is commonly multilevel and includes the velopharynx, oropharyngeal lateral wall, tongue, and epiglottis (VOTE) [5]. Among the VOTE classification, velopharyngeal obstruction is the most common obstruction type and is routinely treated with good results for snoring [6]. In contrast, tongue collapse is difficult to precisely identify, and treatment has variable outcomes [7].

Untreated OSA causes variable complications including neurocognitive impairment and altered cardiopulmonary function [8,9,10]. For patients with OSA, continuous positive airway pressure (CPAP) is considered to be the first-line and gold-standard treatment [11]. However, for varied reasons, many people are intolerant of or unwilling to undergo CPAP therapy and seek surgical intervention as salvage or alternative treatment [12]. Among sleep surgery, uvulopalatopharyngoplasty (UPPP) is the most commonly used procedure for snoring and OSA [13]. However, the low success rate of UPPP for OSA has been criticized for decades [14]. Many studies investigating the outcomes of UPPP showed that persistent tongue obstruction was the main cause of UPPP failure [15].

Tongue surgery includes a reduction in volume and suspension of the tongue base [7]. Previous studies focused largely on reductions in the tongue volume and related changes in adverse sleep parameters [16]. Clinical application of tongue base suspension (TBS) for OSA can be divided into stand-alone TBS and TBS combined with UPPP [17]. A previous study showed TBS combined with UPPP improved the success rate in comparison to stand-alone TBS and as part of a multilevel surgery for OSA [18]. Surgical techniques for TBS included TBS with the Repose^®^ [19] (Repose Surgical Kit, CKA AirVance, Medtronic, Inc., Minneapolis, MN, USA) and modified TBS [20]. For various reasons, Repose is more popular than modified TBS and has gradually become a stereotype in performing TBS. However, there was a recent critical discussion of TBS due to its variable outcomes and expensive suspension kit [21].

**Figure 1:**
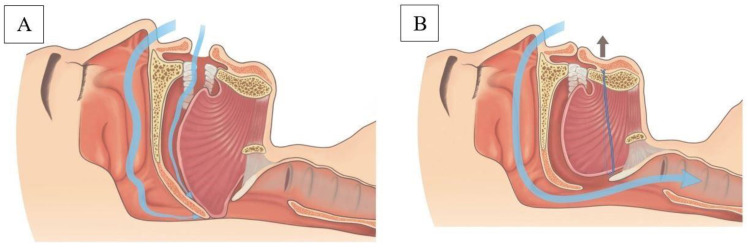
Sketch depicting the proposed change in the hypopharyngeal airflow before (**A**) and after (**B**) transoral tongue suspension.

In this study, we developed a novel technique—transoral tongue suspension (TOTS)—through a sublabial approach to perform suspension of the tongue base and stabilization of the tongue. (Figure 1) We also report on the safety and efficacy of TOTS in treating OSA patients. The results of this study may be helpful in: (1) providing a minimally invasive procedure instead of excision in treating OSA patients with tongue collapse; (2) using an easily performed novel technique without the need of commercialized kits; and (3) avoidance of exterior scars, providing a cosmetic advantage. This study performed a suspension technique that can be used together with a volume-reduction technique in an integrated treatment of tongue obstruction for OSA.

## 2. Methods

### 2.1. Ethics Statement

This retrospective study was approved by the Institutional Review Board (IRB) of the Chang Gung Medical Foundation (number: 202200685B0) and waiver of the participants’ consent. Linkou Chang Gung Memorial Hospital is the main branch of the Chang Gung Memorial Hospitals. The IRB of the Chang Gung Memorial Foundation represents and is in charge of all branches of the Chang Gung Memorial Hospital regarding IRB review affairs.

### 2.2. Study Population

This retrospective study was conducted between September 2018 and April 2021 at the Chang Gung Memorial Hospital, Linkou Medical Center, Taoyuan, Taiwan. Eligible candidates for TOTS were adult patients diagnosed with OSA and were either intolerant of or unwilling to receive CPAP therapy. The inclusion criteria were: age between 18 and 65 years, BMI < 32 kg/m^2^, AHI > 15/h, mouth opening space ≥ 4 cm, tongue obstruction discovered during drug-induced sleep endoscopy (DISE), completed questionnaire (Epworth Sleepiness Scale) [22], and polysomnography before and 6 months after surgery. The exclusion criteria included: significant craniofacial anomaly (syndromic patient), Friedman tongue position [23] IV, combined severe medical disease, previous tongue surgeries and/or radiation therapy over the head and neck region, and high risk for general anesthesia (American Society of Anesthesiologists physical status class [24] >2). Within the study time period, there were 24 consecutive OSA patients enrolled who were mostly (21) middle-aged males with average age of 43 years. The average apnea–hypopnea index (AHI) was 42.2 event/h and the average body mass index (BMI) was 25.7 kg/m^2^.

### 2.3. Surgical Technique of TOTS

**Figure 2:**
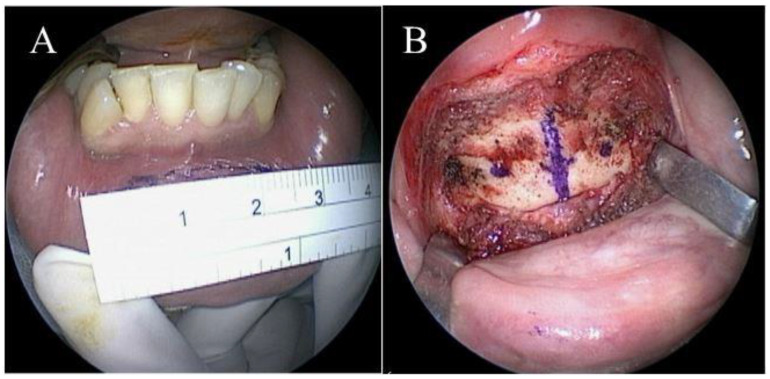
Mandibular vestibular incision (**A**) and marked points for drill on the premandibular plane (**B**).

**Figure 3:**
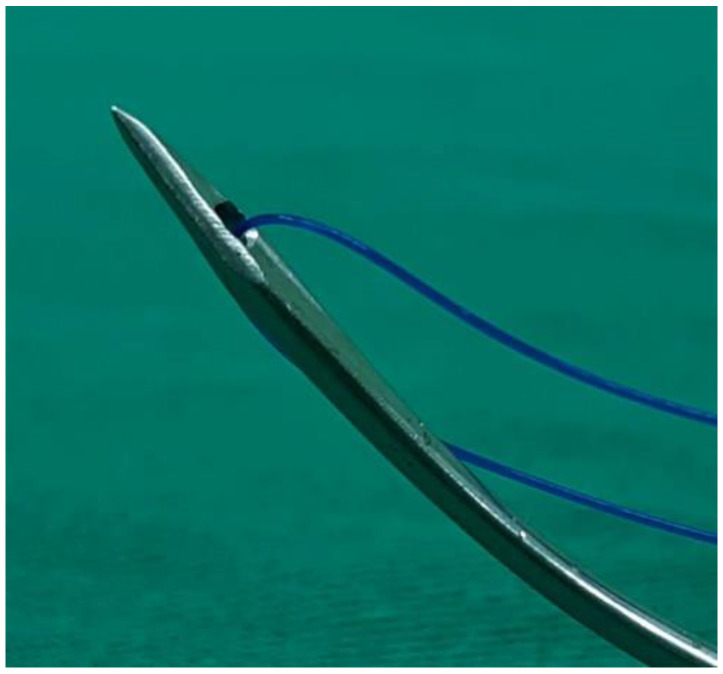
Suture passer attached to a 2-0 interlacing polypropylene suture.

**Figure 4:**
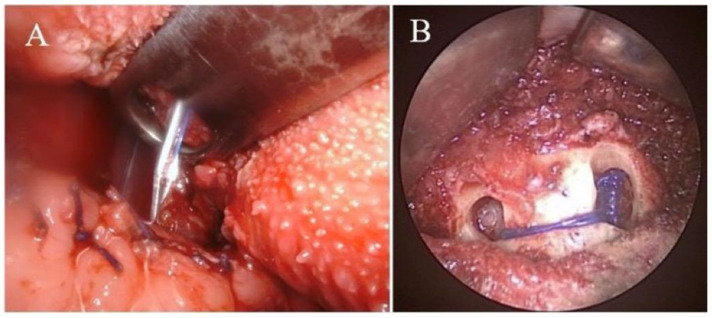
Suture passer emerging from the tongue base (**A**); knots placed inside the hole (**B**).

Under general anesthesia, the patient was placed in supine position with full extension of the neck. The position of operator was in front of the patient. Aqua-better iodine with 50% saline dilution liquid was used to disinfect the oral cavity of the patient. After local injection of 1% xylocaine mixed with 1:100,000 diluted adrenaline at the vestibular area of lower lip, a transverse incision of around 3 cm was made at a distance of at least 1.5 cm from the marginal gingiva of the central lower incisors (Figure 2A). After blunt dissection of sublabial wound, the line of median incisor was marked. Two parallel holes 1 cm from the midline on the mandible were marked (Figure 2B) and then drilled using a cutting burr (3 mm, 60,000 RPM, Medtronic, MN, USA). Afterward, the patient’s mouth was opened via a self-retractor and the tongue was fully pulled out with 3-0 silk to expose the circumvallate papilla and sulcus terminalis. Both sides of the middle third of the sulcus terminalis were identified and marked using electrocautery under a 30° telescope monitor. A suture passer (Obwegeser mandibular awl, Johnson and Johnson, New Brunswick, NJ, USA) (Figure 3) attached to a 2-0 polypropylene (Prolene) suture was used to pass through the right hole, penetrate deep into the mouth floor and tongue, and then emerge from the marked point at the right tongue base. (Figure 4A) A similar procedure was repeated with a 3-0 black silk suture acting a loop at the left tongue base. For safety concerns related to jeopardizing the neurovascular bundle, the emerged passer was kept in the middle third of the tongue base. The polypropylene was then sutured submucosally with a free curved needle to the left marked point and then passed into the loop. The loop and inside polypropylene suture were pulled out together through the left hole. Finally, the two distal ends of the polypropylene suture within the individual holes were tied together and the knots were placed inside the hole to decrease infection and the foreign body sensation in the chin. (Figure 4B) After tightening of the suture, the enlarged retro-lingual air space with a deep dimple on the base of the tongue was confirmed using fiberoptic nasopharyngoscopy. As the final step, multiple interrupted sutures were implemented at the premandibular subcutaneous soft tissue and the vestibular mucosa.

In this study, all patients demonstrated tongue obstruction in independent DISE. Due to the concurrent palatal obstruction shown in the DISE, UPPP was performed simultaneously. In addition, a reduction (ablation) in the volume of the hypertrophic lingual tonsil (grade [25] III-IV) was also implemented using coblation [26] or electrocautery (*n* = 8).

### 2.4. Study Parameters

Demographic data for the patients, including age, gender, body weight/height, and BMI, were collected. Preoperative and postoperative polysomnographic parameters such as AHI and minimal oxygen saturation levels were also recorded. Operation time, hospital stay, and minor/major complications such as bleeding and wound infections were also documented.

### 2.5. Statistical Analysis

Normally distributed data were expressed as the mean ± standard deviation (SD). Statistical analyses were performed using RStudio software (RStudio Team 2015. RStudio: Integrated Development for R. RStudio, Inc., Boston, MA, USA). A *paired t*-test was used to compare continuous data between the baseline and outcome. All *p*-values were two-sided, and statistical significance was accepted at *p* < 0.05.

## 3. Results

### Surgical Outcomes

The operation time for TOTS was around 30 min, and the hospital stay for TOTS with UPPP was 3 days. No wound bleeding or upper airway compromise occurred throughout the postoperative period. Tongue swelling was noted for 1–2 weeks in conjunction with temporary dysphagia and articulation disturbance; even so, all the symptoms were relieved by the first month’s follow-up. Numbness of the lower incisor area was noted in four patients (17%). Sublabial wound dehiscence was found in two patients (8.5%) and healed spontaneously by the first month’s follow-up. There was no change of taste reported at first month’s follow-up.

**Figure 5:**
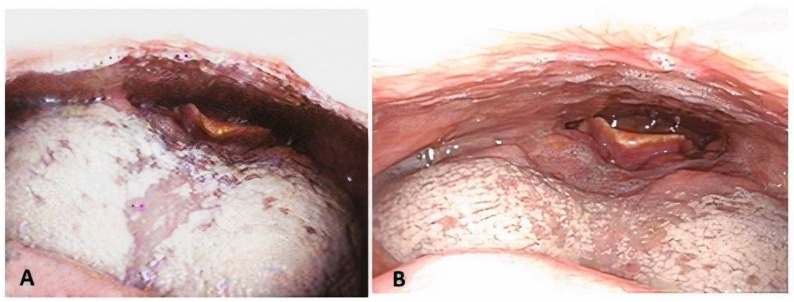
Change of airspace before (**A**) and after (**B**) transoral tongue suspension in fiberoptic nasopharyngoscopy.

**Figure 6:**
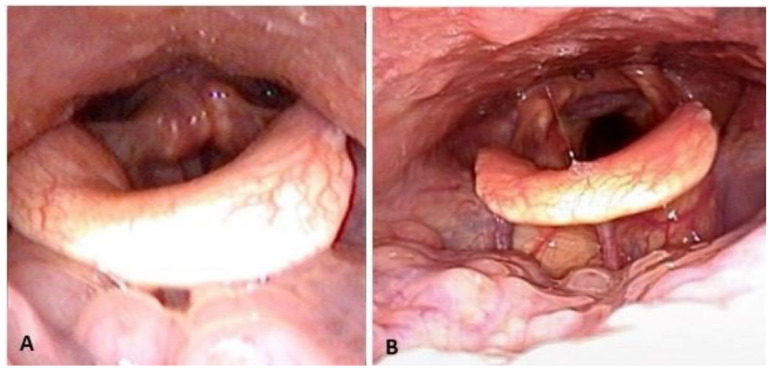
Change of airspace before (**A**) and after (**B**) transoral tongue suspension plus ablation of hypertrophic lingual tonsils in fiberoptic nasopharyngoscopy.

**Figure 7:**
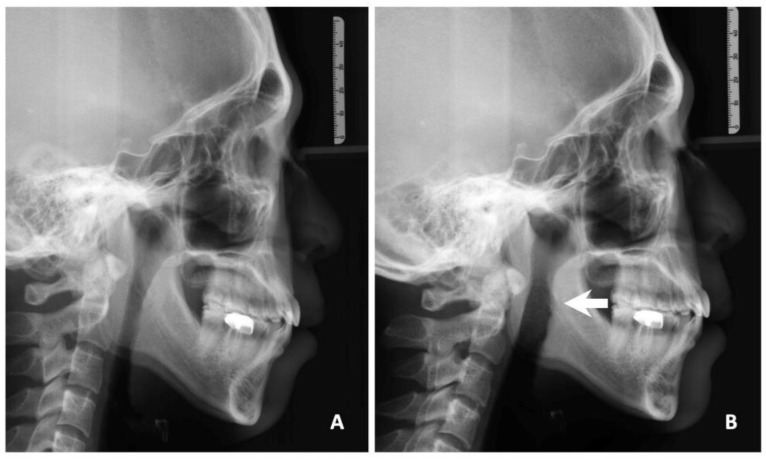
Change of hypopharyngeal space (arrowhead) before (**A**) and after (**B**) transoral tongue suspension plus uvulopalatopharyngoplasty in lateral cephalometry.

**Figure 8:**
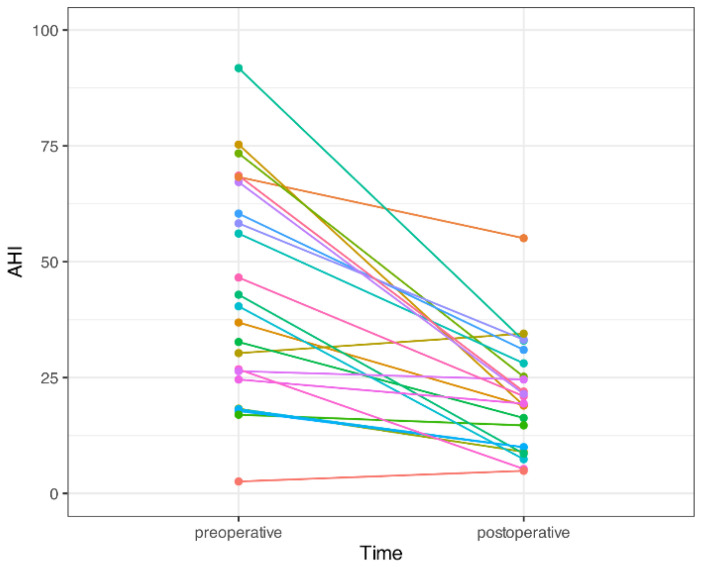
Preoperative and postoperative apnea/hypopnea index (AHI) in individual patients. Different colorful lines in individual patients to obtain a better visual effect.

The quality of life improved significantly in the patients’ subjective daytime sleepiness from the ESS (11.4 ± 3.2 vs. 5.7 ± 1.6, *p* < 0.001). The objective parameters of OSA also improved significantly in AHI (42.2 ± 21.8 vs. 19.5 ± 16.2, *p* < 0.001), minimal oxygen saturation (77.1 ± 12.2 vs. 81.7 ± 8.1, *p* = 0.026), and snoring index (207 ± 141 vs. 101 ± 91, *p* = 0.03) (Table 1). A marked increase in the hypopharyngeal space before and after TOTS was demonstrated using awake fiberoptic nasopharyngoscopy (Figure 5 and Figure 6) and lateral cephalometry (Figure 7). The major outcome in the changes in individual AHIs are demonstrated in Figure 8.

## 4. Discussion

Tongue surgery is always challenging in treating OSA patients with tongue obstruction. This study proposed a novel technique—transoral (sublabial) tongue suspension (TOTS)—to stabilize the obstructive tongue in OSA patients. The results showed minimal morbidities for voice and swallowing in conjunction with significant improvements in subjective daytime sleepiness and objective adverse respiratory events.

### 4.1. Comparison of Previous Techniques

There were various types of tongue surgery for OSA patients, including TORS for resecting hypertrophic lingual tonsils along with redundant, hypertrophic tongue body [27], as well as low thermal devices such as radiofrequency and coblation for tongue-volume reduction without direct surgical resection [26]. However, the enlarged hypopharyngeal space achieved by a reduction in the volume of the tongue base or lingual tonsil did not ensure the stabilization of the tongue during sleep, which turned out to be the variable outcome in adverse respiratory events. Genioglossus advancement has been advocated for decades with modest effectiveness in conjunction with UPPP [28]. Moreover, osteotomy of the mandible can be destructive, and there were risks of hematoma, infection, and damage to lower teeth [29]. Therefore, a minimally invasive tongue base suspension (TBS) was introduced with an aim to stabilize tongue base. A commercial TBS kit (Repose) [19] was introduced through stabilization of the suture over the inner lower gum using a specially designed screw [30]. Later, Medtronic acquired the Repose system (Repose Surgical Kit, CKA Air Vance, Medtronic, Inc.) and the entry of the suspension suture was changed to a submental incision [31]. The Repose system is used worldwide and has become the stereotype for tongue base suspension in OSA despite some individual modifications in the technique [20,32,33].

Inspired by transoral thyroid surgery [34], our team member (Lee, Y.C.) developed the transoral vestibular incision technique and suspended the tongue through the holes in anterior periosteum of the mandible to avoid skin incisions and dissection of the vascular-rich subplatysmal soft tissue. There are four novelties of TOTS in comparison to the Repose tongue suspension. Firstly, TOTS uses a transoral approach instead of the transcervical approach used in the Repose tongue suspension. The transoral approach avoids external skin incisions and resultant potential complications such as granulomas, fistulas, and bleeding [20,35]. Further, the transoral approach offers a superior cosmetic outcome with no exterior scar that is becoming increasingly popularized, as seen in thyroidectomies, when comparing new transoral approaches with conventional trans-cervical ones [36]. Secondarily, TOTS ties the two ends of the suspension string together without the need of a screw. The Repose system uses a screw piercing into the mandible as the anchor to suspend the tongue. The inserted screw as a foreign body increases the potential for infection and a foreign body sensation [18]. Thirdly, TOTS implements two interlacing polypropylene sutures to pull the tongue forward, which not only provides a twofold pulling strength, but also abates the muscle-cutting effect (suture migration) from a single string [19]. Fourthly, TOTS is freely available without the need of commercial kit, which lowers the medical expenditure for patients. In addition, the easily performed TOTS can collocate with coblation [26] or electrocautery to accomplish both the stabilization and a reduction in the volume of the tongue.

Regarding the safety concerns of tongue suspension, previous studies showed varied complications that included delayed local infection, dysphagia/odynophagia/dysarthria, hematoma and edema of the mouth floor, mouth floor cysts, unilateral lingual atrophy and hypoesthesia of the tongue tip, and reduced tongue flexibility [17]. In this study, no major perioperative complications were encountered; temporary tongue swelling was noted in the 1–2 weeks postoperatively, but otherwise, there were no taste changes or swallowing or speech problems in the persistent follow-up after one month. Vestibular wound dehiscents were found in two patients that were healed with oral antibiotic. In general, TBS using a suspension instead of an excision of the tongue base is a low-risk procedure for treating OSA patients with tongue obstruction. Further, TOTS with a transoral (sublabial) approach offers another advantage of a scarless exterior, which has great importance to some patients.

### 4.2. Comparison of Outcomes

Surgical outcome of TBS was variable in different procedures and modalities [17,18]. An evidence-based review revealed that the average preoperative and postoperative AHI was 32.0 and 18.8 (41% reduction) in standalone TBS, with a success rate of 36.6%. Further, the average preoperative and postoperative AHI was 42.0 and 16.6 (62.3% reduction) in TBS plus UPPP, with a success rate of 62.3% [18]. In addition, there was no difference in surgical outcomes between modified TBS and TBS Repose [17]. In this study, TOTS with UPPP achieved a similar outcome in AHI of 42.2 and 19.5 (55.9% reduction), with a success rate of 62.5%. Some factors were related to surgical outcomes of TBS. Omur et al. [20] believed that a low position of the suture and a strong tightness of the suture were the main factors in the outcome of TBS for OSA. Vicente et al. [37] noted that the best response to TBS with UPPP was obtained in patients with a BMI < 35 kg/m^2^. It was noteworthy that we implemented ablation of lingual tonsils for volumetric reduction in hypertrophic lingual tonsil (grades III and IV) before TBS. This method to combine suspension for stabilization of the tongue and volume reduction for widening of the hypopharyngeal airway together to achieve a synergetic effect could be a new concept. In addition, we used two interlacing polypropylene sutures that might have lessened suture migration, which has been presumed as a potential reason for the decline in efficacy for TBS over time [17,19,30,38,39].

### 4.3. Applications

In this study, we reported that TOTS could be used to treat tongue base obstruction in adult OSA patients. When conducting preoperative fiberoptic examinations, clinicians should be aware of the existence of hypertrophic lingual tonsils that may contribute to hypopharyngeal obstruction during sleep. We propose that every adult surgical OSA patient needs to undergo DISE to identify nocturnal tongue base collapse. For patients with tongue base collapse from low muscle tone (endurance) with retro/micrognathia, TOTS is the priority to stabilize the tongue and prevent its collapse during sleep. For patients with tongue base collapse and without the hypertrophic lingual tonsil, TOTS could be considered in the surgical planning of multilevel surgery. For patients with tongue base collapse and hypertrophic lingual tonsil, TOTS could be performed concurrently after lingual tonsillectomy in multilevel surgery.

### 4.4. Limitations

There were several limitations to this study. This study had a small sample size and focused on tongue-obstructed OSA patients, which may not be generalized to all OSA patients. There was also a lack of a matched control group who underwent multilevel surgery without TBS to demonstrate the authentic effects of TOTS for OSA. A comparative study to enroll a matched control group is underway to clarify the orientation and efficacy of TOTS for the time being. In addition, no postoperative DISE was used to elucidate the cause of surgical failure from persisted/residual obstruction(s) in the VOTE classification. In a following study with funding support, surgical-failure patients will receive a repeated DISE with their consent. The short-term outcomes of TOTS may not ensure its efficacy in the long term, since the effects of TBS may decline with time. Regarding this issue, these patients will be followed up regularly for 3–5 years to observe any potential deterioration in clinical symptoms and will be offered a repeated sleep test and any salvage treatment needed. Additionally, assessment of the lingual muscle tone was not performed to identify theoretically good candidates (low muscle tone) for tongue suspension. An extended study using the Iowa oropharyngeal performance instrument is ongoing that will help establish a flow chart for treating OSA patients with tongue obstruction.

### 4.5. Future Directions

For OSA patients with obstruction in the tongue base during DISE examination, we may need to separate these patients into anatomical hypertrophy (obstruction) or physiological dystonia (collapse) and then treat them with tongue volume reduction, tongue base stabilization, or both individually.

## 5. Conclusions

In this study, we reported on a novel tongue suspension technique (TOTS), and the results showed it was scarless, had a low morbidity, was easily performed, was freely available, and was relatively effective in treating adult OSA patients with tongue obstruction. TOTS is feasible as part of multilevel surgery with UPPP in treating OSA. TOTS can also be implemented with lingual tonsillectomy to achieve stabilization of the tongue and widening of the airway. TOTS is less invasive than skeletal surgery and can be an alternative in tongue-obstructed, CPAP-failed OSA patients.

## Figures and Tables

**Table 1:** Comparison of preoperative and postoperative outcomes for TOTS.

Variables	Preoperative	Postoperative	*p*-Value
ESS	11.4 ± 3.2	5.7 ± 1.6	0.0006
AHI (event/H)	42.2 ± 21.8	19.5 ± 16.2	<0.001
Mini-SAT (%)	77.1 ± 12.2	81.7 ± 8.1	0.026
SI (event/H)	207 ± 141	101 ± 91	0.03

Abbreviations: TOTS, transoral tongue suspension; ESS, Epworth Sleepiness Scale; AHI, apnea-hypopnea index; Mini-SAT, minimal O_2_ saturation; SI: snore index.

## Data Availability

The data are not publicly available due to the regulation of our institution and protection of patients’ privacy particular in small sample size group. However, the data presented in this study are available on request from the corresponding author for further research, if available.
